# Genome Sequence of Corynebacterium pseudotuberculosis Strain KM01, Isolated from the Abscess of a Goat in Kunming, China

**DOI:** 10.1128/genomeA.00013-18

**Published:** 2018-03-15

**Authors:** Huafeng Gao, Yuxing Ma, Qingyong Shao, Qionghua Hong, Guoying Zheng, Zhiming Li

**Affiliations:** a Yunnan Animal Science and Veterinary Institute, Kunming, Yunnan, China; b Bureau of Xishan Agriculture and Forestry, Kunming, Yunnan, China

## Abstract

Caseous lymphadenitis (CLA) is an acute, pyogenic, and contagious disease of goat that imposes considerable economic losses for farmers, and it is caused by Corynebacterium pseudotuberculosis. Herein, we introduce the genome sequencing of C. pseudotuberculosis strain KM01, isolated from an abscess of a Saanen goat from Kunming, China. The genome contains 2,198 genes, the total length of the genes was 2,337,666 bp, and the GC content was 52.18%. The number of tandem repeat sequences was 44, the total length of the tandem repeat sequences was 1,970 bp (0.0772% of the genome), the number of minisatellite DNAs was 36, and there were 48 tRNAs and 12 rRNAs.

## GENOME ANNOUNCEMENT

Corynebacterium pseudotuberculosis is a Gram-positive, nonmotile, non-spore-forming, pleomorphic, intracellular, and aerobic-facultative bacterium (1). This bacterium is the etiological agent of caseous lymphadenitis (CLA) disease in small ruminants. Also, this pathogen is a zoonotic agent and may be associated with many diseases distributed worldwide (2–3). The diseases caused by C. pseudotuberculosis result in significant economic loss, because the sheep and goat industries are largely affected in China (4–7). Outbreak of this disease has been expressed in external and visceral forms, either separately or together. In our survey, 10.3% of the goats in Kunming were infected by this bacterium. C. pseudotuberculosis leads to abscess formation abscess formation on the surface of the skin, mostly affecting the head region. External CLA lesions appear initially as abscesses that develop into programmable lesions ranging in size from millimeters to centimeters.

To better understand and enrich the genomic information of a C. pseudotuberculosis isolate from Kunming, China, we sequenced the genome of C. pseudotuberculosis strain KM01. It was originally isolated in Kunming, China, from an abscess in the neck of a 2-year-old Saanen goat in March 2017. A drug sensitivity test showed KM01 to be sensitive to 27 of total 34 antibacterial agents, including cefotaxime, gentamicin, florfenicol, ofloxacin, tetracycline, levofloxacin, azithromycin, ciprofloxacin, acetylspiramycin, erythromycin, chloramphenicol, roxithromycin, sulbactam, enrofloxacin, ampicillin, vancomycin, ticarcillin, penicillin, rifampicin, albamycin, cephradine, cephalothin, lincomycin, amikacin, kanamycin, cephalexin, and clindamycin, but it was completely resistant to sulfamethoxazole-trimethoprim (SMZ-TMP), metronidazole, streptomycin, polymyxin, bacitracin, trimethoprim, and amoxicillin. However, controlling CLA with highly sensitive antibiotics was a total failure, since viable bacteria stay in protected inside abscesses due to the thick capsule that surrounds them.

The genome of C. pseudotuberculosis strain KM01 was sequenced on an Illumina HiSeq 4000 platform after the establishment of a qualified Illumina paired-end library. Raw sequence data were generated by the Illumina base-calling software CASAVA version 1.8.2 (http://support.illumina.com/sequencing/sequencing_software/casava.ilmn). The SeqPrep (https://github.com/jstjohn/SeqPrep) and Sickle (https://github.com/najoshi/sickle) software programs were used to remove contamination reads and conduct read trimming with default parameters to obtain clean data. A total of 9,193,824 clean reads were obtained, with an average coverage of 930.6×. The clean data were used for genome assembly with SOAP*denovo* version 2.04 (http://soap.genomics.org.cn/soapdenovo.html) with multiple k-mer parameters. The GapCloser software (https://sourceforge.net/projects/soapdenovo2/files/GapCloser/) was subsequently applied to fill the remaining local inner gaps and correct the single-base polymorphism for the final assembly results. The assembly produced 5 scaffolds with an *N*_50_ value of 2,337,666 bp. tRNAs and rRNAs were predicted using tRNAscan-SE version 1.23 (8) and RNAmmer version 1.2 (9), respectively. The genome of C. pseudotuberculosis strain KM01 contains 2,198 genes, the total length of the genes was 2,337,666 bp, and the GC content was 52.18%. The number of tandem repeat sequences was 44, the total length of tandem repeat sequences was 1,970 bp (0.0772% of the genome), the number of minisatellite DNAs was 36, and there were 48 tRNAs and 12 rRNAs.

### Accession number(s).

The genome sequence obtained with this study was deposited in GenBank under the accession number CP024995. The version described in this paper is the first version, CP024995.1.
